# Lee's Celsus Translated, &c.

**Published:** 1831

**Authors:** 


Lee's Celsus translated, &c.

Just as we had finished the forgoing article we received the first volume of an edition of Celsus by Alexander Lee, A.M. Surgeon, which is on a very different plan from that of Dr. Milligan. The text which Mr. Lee has followed is that of Targa—some tables and notes are taken from "that splendid and very correct edition by Dr. Milligan"—while he acknowledges his obligations to various ancient and modern authors, in the construction of his work. His principal object was to present the reader "with the most approved text of the various editors from Cæsarius in the year 1528, to that of Targa in 1769," who left little for other authors to do in that respect. The Ordo Verborum, which is of such great assistance to the student, appears to be judiciously constructed; while the translation is rendered as literal as the idioms of the two languages will admit. We shall now offer a specimen of the performance, selected at random, which will convey a very fair idea of the manner in which the work is got up. The chapter which we have accidentally stumbled upon seems to be as appropriate in these days of pestilential dread, as any in the whole book. Neither will the suggestions and precautions of the old Roman be unworthy the attention of the modern Briton.

CAP. X.

Observatio in Pestilentia.

"Est etiam observatio necessaria, qua quis in pestilentia utatur adhuc integer, cum tamen securus esse non possit. Tum igitur oportet peregrinari, navigare: ubi id non licet, gestari, ambulare sub divo, ante æstum, leniter; eodemque modo ungi: et, ut supra comprehensum est, vitare fatigationem, cruditatem, frigus, calorem, libidinem: multoque magis se continere, si qua gravitas in corpore est. Tum neque mane surgendum, neque pedibus nudis ambulandum est, minimeque post cibum, aut balneum: neque jejuno, neque cœnato vomendum est: neque movenda alvus; atque etiam, si per se mota est, comprimenda est: abstinendum potius, si plenius corpus est. Itemque vitandum balneum, sudor, meridianus somnus, utique si cibus quoque antecessit; qui tamen semel die tum commodius assumitur; insuper etiam modicus, ne cruditatem moveat. Alternis diebus invicem, modo aqua, modo vinum bibendum est. Quibus servatis, ex reliqua victus consuetudine quam minimum mutari debet. Cum vero hæc

Ordo.

"Observatio est etiam necessaria, qua quis adhuc integer utatur in pestilentia, tamen cum non possit esse securus. Tum igitur oportet peregrinari, navigare: ubi id non licet, gestari, ambulare leniter sub divo, ante æstum: que eodem modo ungi: et ut est supra comprehensum, vitare fatigationem, cruditatem, frigus, calorem, libidinem: que continere se multo magis, si est qua gravitas in corpore. Tum neque surgendum mane, neque est ambulandum nudis pedibus, que minime post cibum, aut balneum: neque est vomendum jejuno, neque cœnato: neque (est) alvus movenda; atque etiam, si est mota per se, est comprimenda: potius est abstinendum, (quam implere se,) si corpus est plenius. Que item vitandum balneum, sudor, meridianus somnus, utique quoque si cibus antecessit; tamen qui assumitur semel die commodius, tum etiam insuper modicus, ne moveat cruditatem. Modo aqua, modo vinum est bibendum alternis diebus invicem. Quibus servatis, victus debet mutari quam minimum ex reliqua consuetudine. Vero in omni pestilentia facienda sint, tum in ea maxime, quam Austri excitarint. Atque etiam peregrinantibus eadem necessaria sunt, ubi gravi tempore anni discesserunt ex suis sedibus, vel ubi in graves regiones venerunt. Ac si cetera res aliqua prohibebit, utique abstinere debebit: atque ita a vino ad aquam, ab hac ad vinum, eo, qui supra positus est, modo, transitus ei esse."

cum hæc sint facienda in omni pestilentia, tum maxime in ea, quam Austri excitarint. Atque etiam eadem sunt necessaria peregrinantibus, ubi discesserunt ex suis sedibus gravi tempore anni, vel ubi venerunt in graves regiones. Ac si aliqua res prohibebit cetera, utique debebit abstinere: atque ita ei transitus esse a vino ad aquam, ab hac ad vinum, eo modo, qui est positus supra."

TRANSLATION.

CHAP. X. PESTILENTIAL DISEASES.

"There are some things to be observed in a pestilential season, even by a man who is as yet in good health, but yet cannot be secure. At that time it is proper to travel, and to sail: when that is not attainable, to use gestation, gentle walking in the open air before the heat of the day, and unction with the same moderation, and as it has been directed above, to avoid fatigue, crudity, cold, heat, and venery, and confine himself to a strict regimen. If he feel any heaviness about the body, then he is neither to rise in the morning, nor walk barefooted at any time, particularly after meals or the bath: nor to vomit either with an empty stomach, or after supper: nor are the bowels to be relaxed, and if they become loose of themselves, they must be restrained. Abstinence must be observed, if the body be plethoric. Also the bath must be avoided, sweating, the meridian nap, particularly if food have preceded it, at which time food should be taken rather once in the day, and even that sparingly, lest it may cause indigestion: one day water, next day wine is to be drunk, and so on every alternate day. These regulations being observed, there ought to be little or no deviation from the usual diet. But as these rules are applicable in all pestilential times, they must be more strictly adhered to in those, caused by the south winds. The same precautions are necessary for travellers who are about to leave their residences in the sickly season of the year, or when they have arrived in some unhealthy region. But if any circumstance shall prohibit a compliance with all these things, it will be strictly necessary for a person to live abstemiously; and that the transition may be thus:—from wine to water—from this to wine, in that manner which has been directed above."

Only half the work is yet published; and when completed it will be one of the most acceptable presents which can be presented to the student, and to a large proportion of medical practitioners who may wish to peruse the most celebrated medical author of antiquity, with the assistance of a good translation, a correct text, and a "LUCIDUS ORDO."

